# When care hurts: parents’ experiences of caring for a child with epidermolysis bullosa

**DOI:** 10.1186/s13023-024-03502-5

**Published:** 2024-12-27

**Authors:** Elisabeth Daae, Kristin Billaud Feragen, Terje Naerland, Charlotte von der Lippe

**Affiliations:** 1 Center for Rare Disorders, Oslo University Hospital HF, Rikshospitalet, Nydalen, Oslo, 4950, 0424 PB Norway; 2 Institute of Clinical Medicine, University of Oslo, Oslo, Norway; 3 KG Jebsen Center for Neurodevelopmental Disorders, Institute of Clinical Medicine, University of Oslo, Oslo, Norway; 4 Norwegian Centre of Expertise for Neurodevelopmental Disorders and Hypersomnias, Oslo University Hospital, Oslo, Norway; 5 Department of Medical Genetics, Telemark Hospital Trust, Skien, Norway

**Keywords:** Epidermolysis Bullosa, Genodermatosis, Qualitative, Parents, skin care

## Abstract

**Background:**

Epidermolysis bullosa (EB) comprises a group of genetically and clinically heterogeneous diseases characterized by skin fragility and blistering. EB is incurable, and treatment consists of preventing blisters in addition to painful and time consuming skin care, often performed by the parents, in addition to monitoring other symptoms in cases of severe EB.

**Results:**

The purpose of this study was to explore parental experiences of caring for a child with EB. Data were collected from semi-structured interviews, and analyzed through reflexive thematic analysis. The sample consisted of 15 parents. Our analysis revealed three main themes: Becoming a self-taught provider of home-based skin care; Balancing roles; and Ahead of every challenge. The results indicate aspects of caring for a child with EB that may be under-recognized by healthcare professionals (HCPs) and allied caretakers. Examples of this was extensive home care, learning skin care through trial-and-error, tension between illness-demands and the child’s psychological needs, and parents being gatekeepers of their child’s well-being.

**Conclusions:**

Caring for a child with EB may imply practical and emotionally demanding tasks for the parents, and possible unmet healthcare needs. It is important that HCPs recognize and understand the potential burden of extensive home care these parents experience as part of providing for their child with EB and the family.

**Supplementary Information:**

The online version contains supplementary material available at 10.1186/s13023-024-03502-5.

## Background

The skin is the largest of our sense organs, the first organ to develop in fetal life, and the main organ for tactile interaction [1]. Skin-to-skin contact immediately after birth is important for the child and the mother [2], and its positive effects have been well documented in the literature [3]. Caregivers learn when in close physical contact with infants, to more readily recognize their signals—when they are asleep, when they are awake, when they are hungry [4]. This awareness enhances caregivers’ responsiveness to infants. However, if a child is born with the genetic skin disorder epidermolysis bullosa (EB), basic skin-to-skin contact may be a challenge from birth itself [5], significantly reducing the child’s experience with touch [6], and potentially affecting caring practices.

Epidermolysis bullosa (EB) is a group of heterogeneous genetic skin diagnoses characterized by blister formation and mucous fragility, often induced by minimal trauma [7]. EB consists of over 30 subtypes, and is classified into four major categories depending on the layer of skin affected with increasing severity from category 1–3: (1) EB simplex (EBS), (2) junctional EB (JEB), (3) dystrophic EB (DEB), and (4) Kindler syndrome [7]. EB simplex is the most common type of EB, responsible for 70% of all EBs, usually inherited in an autosomal dominant pattern [8]. JEB and Kindler syndrome are autosomal recessive disorders, while DEB is inherited in an autosomal dominant (DDEB) or recessive pattern (RDEB) [8]. Clinical manifestations range from relatively mild skin reactions, to severe forms with a lethal outcome within the first year of life [8].

Although gene therapy is promising for EB, there is no curative treatment [9]. Treatment consists of minimizing the risk for blisters, draining of blisters, wound care, changing bandages, lubrication of the skin, pain relief, and in more severe cases also involves potentially dealing with consequences of pseudosyndactyly, osteoporosis, anemia, esophageal strictures, and other disease complications. Parents are often in charge of the children´s daily care, including wound care, which may be time consuming and painful to the child [10, 11].

Pediatric chronic illnesses affect the child and their entire family [12, 13]. The parents are usually primary providers of medical care [14] and may experience a higher number of caregiver challenges than parents of healthy children [15, 16]. Adapting to their child’s diagnosis and care needs becomes essential for minimizing parents’ and children’s emotional distress and facilitating coping efforts [17].

Gowran et al. [18] state that “*Living with EB and supporting people with EB goes much more than skin deep*”, mirrored in research of parents caring for a child with EB. Quality of life and physical and emotional well-being seem to be negatively impacted in parents caring for a child with EB [19], because of time constraints due to skin care, dressing changes, and the financial burdens of associated medical costs. Other studies highlight that skin and wound care has a high emotional impact on the parents as well as on the children [10, 11, 20–22], especially for the more severe forms of EB [23, 24]. Raising a child with EB may also affect parental relationships, again with a higher impact for more severe EBs [25]. A scoping review found that the most common support needs among parents raising a child with EB were emotional, followed by practical, social, and physical needs [26].

Another recent review declared that there is a paucity of studies focusing on the families caring for children with EB [27]. With this backdrop, our study aimed at exploring first-person experiences of parenting a child with EB, with a specific focus on how parents experienced long-term skin care administered at home, and the impact of EB on the family and daily life.

## Methods

### Ethical considerations

This study was conducted in accordance with the Helsinki Declaration. Ethical approval for the study was obtained from the Ethics Committee (South-East Norway, reference number 2019/567), and was accepted by the Oslo University Hospital’s Data Protection Office (reference number 19/08354). All parents gave written consent to participate. In addition, individuals with EB who were 16 years and older provided written consent for us to invite their parents to participate. To protect privacy and maintain anonymity, information regarding the gender, exact age, and the number of children with the same diagnosis in a family is not provided, and we refer to the diagnoses as severe or mild EB without elaborating on the precise EB diagnosis.

### Study design

We used a qualitative explorative approach with a semi-structured interview guide which allowed a systematic exploration of topics using predetermined open-ended questions, participant-led exploration, and encouraged two-way communication [28].

### Recruitment

The Center for Rare Disorders (CRD), Oslo University Hospital, (Norway) is a national multidisciplinary resource center for several rare disorders, including EB. CRD provides knowledge and competence on rare disorders to individuals/families with rare disorders, health care professionals (HCPs) and the general public. CRD does not provide medical or psychological treatment. CRD has a patient registry, including patients or parents who have consented to registration. At the time of this study, the registry included 27 individuals with EB under the age of 16 and 45 individuals 16 years or older. The parents of children with EB (*n* = 27) who were under 16 years of age received an invitation by mail to participate in the study in August, 2019. A reminder letter was sent in October, 2019. In addition, 45 letters were sent to individuals with EB born between 1980 and 2003 requesting their consent to invite their parents to participate in the study. Social media, the Department of Rheumatology, Dermatology and Infectious Disease, Oslo University Hospital, and the national Debra patient organization also provided information about the study, encouraging parents to participate.

### Follow-up of patients with EB in Norway

People with severe forms of EB are followed and treated throughout life by an EB-team at Oslo University Hospital (OUS), consisting of nurses and medical doctors. Medical competence includes dermatology as well other specialties, such as anesthesiology and pediatrics, in addition to psychologists, physical therapists, social workers, and nutritionists, who cooperate closely with the EB-team. The EB-team provides an outpatient clinic for EB-patients at OUS. Children with chronic pain due to EB, are followed by the outpatient clinic, a palliative care team in OUS, or receives advanced hospital treatment at home. When a child with EB is discharged from hospital, the primary health service in the municipalities is responsible for the follow-up of the child and the family. For the severe forms of EB, the local follow-up is provided in collaboration with the EB-team. Therapeutic parent education is provided before discharging the child, as well as during follow-up visits.

### Data collection

The semi-structured interview guide was informed by previous literature and developed by three of the authors (ED, CvdL & KBF) and refined after inputs from the patient representatives. The interview guide (Supplementary material appendix 1) consisted of open questions, such as parents’ experiences of: pregnancy and birth, the initial time after birth, the diagnostic process, touching the baby’s skin, cuddling with the baby, and skin care and how it evolved as the child grew older. Follow-up questions were asked to clarify answers or seek elaboration upon responses. The interviews were conducted by ED from January to August 2020. Three of the interviews were conducted face to face. Due to the Covid-19 situation, the remaining interviews were conducted by phone. On average, the interviews lasted for 63 min (range: 31–95 min), and were audio-recorded and transcribed verbatim. Prior to each interview, the first author explained the study purpose and regulations regarding confidentiality, and specified the participants’ rights to withdraw their consent at any point.

### Data analysis

We used a reflexive thematic analysis with the six phases outlined by Braun and Clarke [29, 30] to identify themes/patterns within the data set (Table 1). First step involved that the first author read all transcribed interviews, to refamiliarize herself with the context of each interview and the content of the dataset as a whole. The interviews were coded on a semantic level (inductive data-driven codes using the participants’ own words). ED and CvdL coded the interviews individually to ensure credibility and dependability, before analyzing the data together and reaching consensus about the codes. ED, CvdL and KBF interpreted emerging patterns and examined the underlying ideas and assumptions, and identified clusters of codes to represent patterns in the data. The analysis process involved making sense of the relations among the themes and subthemes, which were created. All authors checked the adequacy and suitability of the themes.

**Table 1:** Stages of the analysis

Stage 1	Verbatim transcriptions of the interviews were done, and transcripts were read and re-read. Initial thoughts were written down. ED and CvdL
Stage 2	Inductive coding of all interviews were conducted by hand. The initial codes were defined broadly to bring together a group of data excerpts that could be related, and sticky notes were used as tools. One document with all the codes was made for each transcript, in order to gain an overview of the codes, both within and across interviews. ED and CvdL. Meetings with all four authors took place to discuss main tendencies within the datasets.
Stage 3	We searched for themes within and across interviews, and mindmaps and color codes were used. ED and CvdL.
Stage 4	The themes were reviewed and named, and all the relevant data for each theme were gathered. All authors.
Stage 5	Themes were defined, as well as how they related to each other. All authors.
Stage 6	Writing up the results. All authors.

Hill et al. [31] have suggested the frequency-labels “general,” “typical” and “variant” to indicate the representativeness of findings and the reappearance of themes. To adhere to Hill et al. [31]’s terminology, the label “general” is used to include all but one of the cases and is referred to as “all” participants. “Typical” included more than half of the cases and is referred to as “most” participants. “Variant” included at least two cases and is referred to as “some” participants.

### Reflexivity and trustworthiness

Reflexivity was emphasized throughout the data analysis [32]. ED, CvdL and KBF are all familiar with EB. ED and CvdL have worked in CRD through many years, and have met children with EB and their parents in different settings (their homes, their schools, the hospital and courses/seminars). KBF has theoretical knowledge about the disease and what it is like to live with EB or raising a child with it. This expertise helped identify areas in the interviews that merited further probing, and was useful during the analysis to recognize parents’ experiences. However, prior knowledge and expectations may also potentially bias the interpretation of data. The third author of this article, TN, is unfamiliar with EB, but familiar with other rare disorders and is an experienced research psychologist, and was an important discussant. Our different backgrounds encouraged reflections mirroring our positions and experiences. To enhance the trustworthiness of this study, four steps were taken [33]: Credibility checks were performed during each interview. At the end of the interviews, the participants were asked whether there was anything important to them that had not come up as a topic or a question during the interview. Member checking was carried out by discussing the findings with the study’s reference group, consisting of two parents, and they were encouraged to assess whether the results captured the essence of the parents’ everyday life with regard to caring for a child with EB. Further, investigator triangulation was used to confirm findings and different perspectives as described in the section of data analysis [34]. Excerpts from the interviews are presented to provide transparency in the analytical process and ensure the credibility of the results. F represents “father,” and M indicates “mother.”

## Results

### Sample characteristics

A total of 15 parents (11 mothers and four fathers) of 20 individuals agreed to participate in the study. For two families, both parents agreed to attend. Seven of the children had severe EB (RDEB, JEB), and 13 had a mild form (EBS, DDEB). A total of 12 were below the age of 18, and eight were older.

Our analysis yielded three main themes: Becoming a self-taught provider of home-based skin care; Balancing roles; and Ahead of every challenges. Table 2 shows the main themes and subthemes.

**Table 2:** Main themes and subthemes

Main themes	Subthemes
Becoming a self-taught provider of home-based skin care	Interpreting the child’s symptoms and pain Organizing skin care and experiences with home care providers Knowledge transferred from parents to child Fear of social misinterpretations
Balancing roles	Tension between illness-demands and the child’s psychological needs Siblings and parental role
Ahead of every challenge	Parents as gatekeepers Inclusion and exclusion

### Becoming a self-taught provider of home-based skin care

Children with EB usually develop blisters and wounds before they are able to talk and express themselves. All parents used all of their senses to identify symptoms of EB, being attentive to their child’s body language at all times, creating meaning from the child’s nonverbal cues. At the same time, parents constantly monitored how their caring behavior affected the child, making adjustments when necessary. All parents had to organize the extensive skin care at home with or without help from health care professionals such as home care nurses. As the child grew older, most parents would transfer their knowledge about EB and skin care to their children as part of increasing the child’s autonomy. Parents actively informed others about their child’s diagnosis, in order to avoid misinterpretations regarding the child’s symptoms.

### Interpreting the child’s symptoms and pain

This subtheme is closely related to parents’ lack of knowledge about EB. Most parents, irrespective of EB severity, experienced receiving insufficient information from HCPs on how to deal with skin care in everyday life. Therefore, they mostly had to find out how to deal with the child’s symptoms on their own.

Before the children could express themselves verbally, parents had to translate signals of discomfort and learning to differentiate among different types of crying. Intensive crying often signalized new wounds or blisters that needed draining, and they often had to untie bandages to localize them.

*“I knew the difference between crying because of pain and other kinds of crying. I don’t know how*,* but I just knew when something wasn’t right. I kind of got this feeling”* (M, severe EB).

Part of this learning process was through trial-and-error. Communication between child and parents improved as the parents learned to master several illness-related caregiver skills.

Most parents believed their child experienced pain during skin care, and when new blisters appeared and healed, but some recalled that they perhaps had not been fully aware of the child’s pain.

*“I wasn’t aware of pain back then [when the child was an infant]*,* but when I think of it now*,* she could definitely have experienced pain. She cried a lot during the day*” (M, severe EB).

The infant’s physical vulnerability and parents’ fear of inducing blisters and wounds was described as hampering the emotional attachment between the parent and child:

*“Maybe we deliberately postponed becoming attached to her… we kept her at a distance. You cannot build the same relationship straight away*,* that other parents are able to”* (F, severe EB).

A few of the children with severe EB also developed blisters inside the mouth, throat and esophagus. Crying could result in internal blisters. Therefore, parents strived to prevent the baby from crying, which was exhausting and led to apprehensiveness:

*«Everything was difficult when we came home from the hospital. When he was admitted*,* we slept a lot. At home we couldn’t sleep*,* we constantly watched over him. We still don’t sleep well*,* something happens all the time”* (F, severe EB).

Interpreting the child’s nonverbal way of communicating remained important even when the child had developed the ability to express itself verbally. Limping in the child could for instance indicate blisters under the child’s feet, even though the child had not voiced this explicitly.

### Organizing skin care and experiences with home care providers

A few parents, all of them raising a child with severe EB, reported spending several hours with skin care:

*“Hours went by with taking care of her skin*,* sometimes half a day. In a way*,* skin care was all there was. I never did anything but taking care of her skin when she was little. I didn`t leave the house for a very*,* very long time“* (M, severe EB).

Some of the parents needed professional help with skin care as they found it emotionally hard and time consuming to carry it out by themselves. They were offered help in their homes, provided by public HCPs. A few parents expressed frustration about lack of coordinated care with several different nurses coming to their home, and the challenge with nurses only visiting mornings and evenings while the parents felt they needed help also during the day. Some parents struggled to perceive external care support as helpful, and feared that the nurses’ lack of knowledge about EB would be detrimental to the child.

*«The nurses who came*,* did not manage to do anything. In a way*,* you do everything yourself*,* or the skin will worsen. It was hard”* (M, severe EB).

### Knowledge transferred from parents to child

Most of the parents raising a child with EB, reported helping the child to gradually take on more responsibility of skin care and the consequences of living with EB. By doing this, they tried to unify their own acquired knowledge of the disease with the child’s experiential knowledge of how it is to live in a body affected by EB. The children needed to learn to interpret their own body symptoms and signs; their lived experience of EB, the blistering, and pain. Most parents stated that as the child with EB grew, they tried to involve them in decision-making processes regarding skin care, dressing, and socially interaction with peers, to gradually enhance and empower their readiness for self-care. This was a more conscious process in families raising children with mild EB. Parents invited the child to cut bandages and apply moisturizer, and explained what they were doing during skin care; hence the children developed an understanding and a vocabulary for the procedures they would need to know, as they grew older. The responsibility and skill of handling blisters was gradually transferred, by encouraging the child to pop and drain blisters when experienced by parents as mentally ready, such as when the child started to ask questions about practical aspects of skin care:

*“It came to a point where he wanted to do it himself*,* because it felt safer to pierce a blister himself rather than letting someone else do it. When he was mature enough motorically*,* it was this natural turning point”* (F, mild EB).

### Fear of social misinterpretations

A few parents feared other people would assume they harmed their child when it screamed during skin care.

*“We were on vacation*,* she was about three years old*,* and had lots and lots of blisters. We stayed at a hotel with thin walls that you could hear everything through. The blisters needed to be punctured. She screamed and protested while we took care of her skin. We punctured the blisters*,* packed our bags*,* and left the hotel immediately afterwards”* (M, severe EB).

Other parents perceived questions from others as intrusive. A few parents had been asked if their child had burn damages, while others had experienced being accused of not looking after the child properly due to the appearance of the skin. Hence, parents felt obliged to disclose diagnostic information in social settings, to prevent misunderstandings and raise awareness of EB.

### Balancing roles

All parents described difficulties in combining the parental role with the care worker role of treating their child at home. The impact of EB on the family and unaffected siblings was also challenging.

### Tension between illness-demands and the child’s psychological needs

Skin care in young children, unable to understand verbal language, and hence impossible to prepare for painful procedures, were emphasized to be the most stressful and challenging task for parents as caregivers. They were afraid the child would feel unsafe at home with them, because of unpredictable skin care. Most parents of young children, irrespective of disease severity, stated that they had to use coercion in order to keep the child still when performing skin care, and needed assistance from the other parent in these situations. Some reported it felt like abuse when forcing their child to lay down or sit still during skin care procedures. It was difficult for the parents not to be able to accept the child’s boundaries and efforts of making them stop hurting them:

*“We did the skin care ourselves*,* with lots of screaming and crying. I had to hold her hard to keep her still. (…) it felt abusive*” (F, severe EB).

This created an emotional ambivalence in the parents, torn between what they had to do to help their child (skin care) and what they wished they could (avoid skin care and help calming them down). Day-to-day concerns and illness-management demands induced uncertainty and apprehensiveness in parents, and could get in the way of attuning to the child’s needs and emotions in appropriate and supportive ways, even when the parents did identify what the child required. They experienced a challenge in combining the dual roles of loving caregiver and informal clinician. Other parents, irrespective of disease severity, reported that their child feared pain. When the child observed the needle in their parents’ hand, the child knew what lay ahead, and started crying and protesting.

Most of the parents struggled to find a balance between letting the child explore the surroundings and build autonomy versus how strict the restrains imposed by the parents should be.

### Siblings and parental role

Some of the children with EB had older siblings. For a few, their first experience with the new sibling was in the hospital:

*“He was so sad*,* he had waited for his sibling. He felt so miserable*,* going home without her»* (M, severe EB).

The parents felt apprehensive, and the baby’s diagnosis was unclear. One or both parents had to stay at the hospital with the newborn. Hence, the unaffected sibling had to cope with his own stress without one or both of the parents, making the parents feel torn between their children’s needs.

A few parents experienced going home from hospital as rough on the unaffected siblings, who had to witness distressing procedures, as well as stressed and sleep-deprived parents.

All the parents experienced that the unaffected siblings had to take the child with EB’s needs into account all the time. Time consuming skin care tied the parents to the child with EB, which meant less time with the unaffected sibling(s).

*«The poor brother had to wait all the time. Wait until we’re finished in the bathroom*,* wait*,* wait*,* wait…”* (M, severe EB).

Some parents also worried about sibling rivalry because the child with EB usually was prioritized, and they were worried that the child with EB could be jealous of the unaffected sibling. However, most parents stated that the sibling relationship turned out to be characterized by loving communication and interaction.

Unaffected siblings had in some ways experienced restrictions and/restrains, irrespective of disease severity. The parents wanted family activities to include everyone, but in order to protect the child with EB, family activities did not involve much physical activity. For many ethnic Norwegian families, everyday life consists of outdoor activities which were difficult to combine with parenting a child with EB. Hence, unaffected siblings often missed out on outdoor activities with their families, and finding a balance between the children’s needs was a daily struggle:

*“We have tried our best*,* but there will be limitations anyhow*,* since finding an activity that include them both is impossible”* (M, mild EB).

### Ahead of every challenge

Being a parent to a child with EB involved several challenges beyond extensive homecare. The parents reported being gatekeepers of the child’s surroundings in the broadest sense, being ahead of several practical and emotional challenges in everyday life. Most parents stated that EB required a great extent of planning and coordinating, for the child to be able to participate in social activities.

### Parents as gatekeepers

As the infant grew older, parents reported having adapted to a life with EB. Some parents stated that the most challenging part of raising a child with EB was the restrains posed on the child due to EB, such as depriving the child of experiences to prevent blisters from occurring.

A few parents of children with severe EB, reported that it took a great deal of planning to use common things like a car seat, a child chair or a pram, since everything needed adjustments like padding, to avoid blisters and wounds:

*“Nothing is ready for use. You have to reflect on everything*,* and if you take some things into account*,* there will be other things you didn’t consider*,* that go wrong. You must try and fail at absolutely everything. I can’t buy her anything I don’t need to improve in one way or another”* (M, severe EB).

A few parents of children with mild EB, reported helping their child to realize that they would not be able to choose education or occupation freely, due to the limitations EB posed on them. They felt obliged to tell their child they should avoid choosing a physically demanding job, as this would probably lead to incapacity to perform the job. This had been an ongoing dialogue between the child and the parents, and led to a feeling of loss and sorrow. A few parents had kept their worries about restricted occupation possibilities, without discussing it with the child. Parents of children with severe EB did not raise this issue.

### Inclusion and exclusion

Most parents shared that EB had a social impact on their child. Parents of children with severe EB, arranged visits at their own house instead of encouraging their child to visit others, because it was easier to take care of potential skin damages or blisters at home. Social inclusion was important to all the parents. Hence, to enable the child to attend birthday parties, for example, parents decided to attend as well:

*“It is ok for her that we attend birthday parties. We have to help her keep an eye on what is happening around her. She can’t attend to all activities but at the same time*,* it’s important for her to be there”* (M, severe EB).

All the parents reported that playing sports was a problem for their child, irrespective of EB severity. Contact sports were particularly challenging, and being unable to participate created a feeling of exclusion:

*“It’s socially inhibiting. Once they turn 12–13*,* and there are 3–4 football trainings a week and football cups*,* they get lots of pain*,* and can’t attend”* (F, mild EB).

The parents also felt they were not free to travel wherever they wanted. Most parents stated that holidays were a challenge. Travel to warmer regions was difficult due to more blistering when exposed to heat. Holiday planning also needed to include as little walking as possible. Long flights were also a problem to children with severe EB.

## Discussion

The purpose of this study was to explore the experiences of parents caring for a child with EB, especially how parents experienced long-term skin care administered at home, and how EB impacts the family and daily life. The results indicate that parents faced multiple challenges.

### Impact on parent-child relationship

The parents described challenges in balancing the dual roles as informal health practitioners and loving parents, which extended beyond skin care. Disease management was not experienced as just a clinical task. Parents had learned through carefully monitored trial-and-error about EB and its symptoms by observing their child throughout the years, and hence their knowledge extended the biomedical knowledge of the disease. The role as informal health practitioner also encompassed both their own emotional distress as well as the distress parents caused to their child. This dilemma appeared during skin care when the parents identified what the children with EB needed, but due to the illness-demands, were unable to fulfill the natural parental instinct to protect and comfort the child. The power struggle between child and parents induced by skin care, created guilt in parents. Most parents of children with severe EB in our study feared touching the child as it could induce blisters. One may therefore ask whether skin care and the child’s reaction to it, may affect the parent-child relationship. Results from our study indicate that the nature of EB may affect early emotional relationships, in concordance with other studies [6, 19].

All parents, raising children with and without EB (siblings), were aware that the child with EB received more attention and time with the parents than the healthy siblings. The parents’ perception of family cohesion was also challenged, because it was difficult to do something with the whole family together. Parents did not seem to know how non-affected siblings had perceived their upbringing, indicating that parents possibly found it difficult to have conversations with their children and siblings about emotional reactions to the family’s situation, as reported in other studies [35, 36]. There is a paucity in research on how parents experiences communicating with their adolescent about their visible difference, because they may find it difficult to raise appearance-related issues, feeling insecure about how and when to address such issues, and which words to use [37]. There are reasons to assume that siblings may be negatively affected by the time skin care requires and the limited possibilities of doing things together as a family [38].

#### Distrusting HCPs in municipalities with restricted knowledge about EB

Despite demanding treatment-related experiences, some parents found it difficult to accept help from HCPs coming to their homes to assist with skin care. Parents had used time and all of their senses to learn to interpret their child’s behavioral cues, and did not trust HCPs or others offering practical support, to understand the child’s needs and symptoms. Parents felt responsible for their child’s quality of care, and did not trust the nurses’ qualifications of treating a child with EB. The lack of knowledge about EB in HCPs mirrors van Scheppingen et al. [11]’s findings. Parents’ mistrust in HCPs may result in avoiding professional care even when it would be necessary [39], which may have consequences for the child’s health care.

#### Protective vigilance or overprotection

Complex management of EB affected most of the child’s and the family’s domains of life, in line with previous research [11, 27]. One coping strategy used by parents has been described as “protective vigilance” [40], intervening pro-actively in an effort to alleviate possible stressors for the child, as was evident also in our study. The role as informal health practitioner expands into the parents’ gatekeeper function, inducing a kind of watchfulness and attentiveness. The gatekeeper function could be considered a way of trying to maintain some degree of control. Worrying about what could happen if not paying enough attention or not taking the right measures, was reported by all parents in our study. Parents stated an ambivalence between teaching their children to be attentive to their body’s symptoms and signals and to be alert to potential threats in the surroundings, while at the same time wanting the child to be able to attend social gatherings. Parenting a child with EB means fluctuating between living a normal social life and take EB into account, a challenging act of balance affecting the entire family.

Care-giving demands in EB are immense, and increased levels of parental protection might be adaptive as parents strive for maintaining their child’s health and well-being, and control their own experience of distress. However, excessive protection, can come in the way of the child’s developmental stage [41]. The right balance between witnessing the child’s distress, and parental overprotection may be difficult to find in families with EB [42]. The parents in our study could not protect the children from pain, and therefore tried to protect the child from developing blisters instead. Restricting the child’s movements and choices, due to parents’ anticipatory anxiety, could be interpreted as parental overprotection. It is also plausible that parental overprotection increased the child’s apprehensiveness. One could ask whether it could be confusing to the child how parents protected them in every sense, except from in the situation they really wanted protection, during skin care.

Prior to healthcare transition, there must be a process at home in which the parents help and prepare their children to adhere to self-management of the disease. There is a paucity of research in this area [43]. A challenging period of time for parents raising a child with EB, is the complex shift of management responsibility from parents to adolescents, both parts needing to adjust to the changes in their roles. Parents need to allow adolescents to develop technical self-care skills and their own knowledge of EB, a process that seems to induce parental anxiety and uncertainty related to how much responsibility they can transfer to the adolescent. This parental struggle is often due to fear of worsening health outcomes.

In our study, parents of children with mild EB talked about the process of transferring health (skin) care to the child for them to reach autonomy, while parents of children with the most severe subgroups did not elaborate on this issue. One explanation could be that children diagnosed with more severe EB, are prone to fusion of fingers [44], which could affect fine motor skills and make it more difficult for them to perform skin care themselves. Due to this, this subgroup will most likely rely on help also after reaching adulthood, and parents may therefore not have the same focus on self-management. Another explanation could be that skin care in children with the severe subtypes is even more time consuming and emotionally draining than for milder forms of EB, and parents may therefore have little time or energy to reflect upon this issue.

Transition from pediatric to adult care for patients living with EB has received some research attention [45, 46]. In order to help adolescents with EB with a successful transition from pediatric to adult care, interdisciplinary collaboration is needed [46]. The intimate nature of skin care may challenge the child’s boundaries, as shown in a qualitative study of another skin diagnosis involving daily skin care [47]. To gradually enhance and empower the child with EB’s readiness for self-care, HCPs should include the children in decision-making processes regarding skin care, as well as helping parents in this task. Parents in our study came across as informed, competent, and experts on their child, reporting always present concerns for their child’s well-being. Perhaps the greatest challenge was to balance between fostering the child’s growing independence while also securing their own and the child’s wellbeing. In Norway, an additional challenge in transition could be that the EB-team treats and follow-up all people with severe EB throughout life. This could potentially make it more difficult to encourage autonomy, since EB-team members and parents know each other very well, which can complicate setting boundaries.

#### Pediatric medical traumatic stress

All parents reported that their child experienced pain, especially during skin care, which is a common and burdensome daily routine for most people with EB [48]. It is well documented that wound care is closely linked with anxiety in children with EB [11, 48–50]. Therefore, children with EB, unaffected siblings, and their parents may be prone to experiences of pediatric medical traumatic stress (PMTS), defined as: *“the psychological and physiological reactions of children and their families to pain*,* serious illness*,* medical procedures*,* and invasive treatment experiences*” [51]. PMTS is a set of symptoms, including arousal, re-experiencing, and avoidance that might be present with or without meeting clinical criteria for a PTSD diagnosis [51]. PMTS may have an important and unrecognized impact on parental well-being [51]. Our study has not specific data on this, but a contributing factor to PMTS in families with EB, may be that parents are the ones to inflict pain in the child. Taking care of symptoms and trying to avoid them was an inevitable source of emotional distress to the parents, and could be characterized as ongoing exposure to stress and trauma. Stress reactions to medical events are normal, but if reactions persist and disturb the child’s or parents’ everyday functioning, they warrant attention and in some cases, intervention [52]. Importantly, the occurrence of traumatic stress reactions in children or parents is more closely related to the person’s subjective experience of the event, rather than any objective medical criteria [51]. Our study, as well as previous research [11, 25, 50, 53], indicate that parents of children from all subgroups of EB, experienced the child’s pain and anticipatory anxiety as emotionally draining and stressful. It is also described that it was more common that children became silent or hid their emotions during skin care than expressing emotions [54]. Hence, healthcare professionals working with children with EB, irrespective of disease severity, should aim at identifying families at risk of developing traumatic stress reactions, and help patients, parents, and their siblings with coping by making appropriate referrals. To try to minimize trauma such as pain during skin care, HCPs should focus on pain management as early as possible, in order to reduce the negative impact of EB on the affected children and their parents [19, 55].

### Implications for practice

When a child is born with EB visible from birth, it is important that a well-trained multidisciplinary team takes care of the infant and its family [56]. Proper pain care is especially important. Parents need to understand the disease and its consequences, and will have to learn many medical procedures and concepts. As a consequence, cuddling and ordinary “baby-care” may come in the background. Psychological support is important, to help both the child and the parent with attachment and bonding, possibly through other means than touching.

Parents need to learn how to prevent and help rapid healing of wounds by the use of non-adherent bandages/dressing, nutrition and infection control, and the EB-team should be in charge of this. Solutions with home-care nurses should be flexible, tailored to the families’ needs. HCPs assisting parents in skin care at home, should be educated on EB and its psychological impact, at dermatologic wards before the child is discharged, preferably including the family to create trust.

Parents need help to cope with their infant’s disease and everyday life. Medical care should be focused on trying to minimize traumatic stress. HCPs should focus on using trauma-informed care which aims to identify stressful reactions, consider how treatment experiences impact patients or parents, and consider the need for psychological interventions [49, 51]. Parents might need help in setting boundaries regarding how to let the child explore its surroundings, and how they may help their children in making appropriate choices when they get older. Children also need to be educated about EB and how the disease may impact their lives. Helping the children to adapt among peers should also be highlighted. HCPs working with children with EB, irrespective of disease severity, should aim at identifying families at risk of developing traumatic stress reactions, and help patients, parents, and their siblings with coping by making appropriate referrals.

Routines and procedures should be developed in relation to the child’s age. HCPs should help parents and children to cope with overprotective parenting practices, and to ease the shift of self-caring practices and development of autonomy [57].

### Limitations and strengths

This study was performed retrospectively. EB comprises varying phenotypic presentations, and it is important with a heterogeneous sample to capture the range of their experiences. Because of this, we invited all parents of children with EB in the database of Center for rare disorders. A limitation could be that data consisted of the parents’ recollection of events that took place ranging from 2 to 37 years prior to the interview, and the data collection was based on the parents´ experiences only. Employing multiple data collection methods could have enhanced validity of our study [58]. On the other hand, data did not seem to indicate differences related to whether experiences were more or less recent.

The number of participants could be a limitation, but EB is a rare diagnosis, and the Norwegian population is small. Our sample is diverse with representation of cultural and ethnical minorities, first of all for the severe subgroups of EB. A subgroup of parents had another mother tongue than Norwegian, but were not interviewed using their mother tongue, which may have restricted their ability to share personal experiences and in in-depth communication. At the same time, severe autosomal recessive disorders, like for instance dystrophic and junctional epidermolysis bullosa, is overrepresented in cultures practicing consanguineous marriage [59, 60]. The present study’s strength is therefore giving voice to their experiences of raising a child with EB, increasing diversity in healthcare research.

A strength of this study is that we are four authors with different backgrounds, and ED, KBF and CvdL have experience in qualitative research.

## Conclusion

Our results indicate there may be aspects of caring for a child with EB that can be invisible to HCPs and allied caretakers. EB takes a variety of forms, with different symptoms and severity, and therefore there are consistent demands and burdens for all caregivers dealing with EB. A consequence of this may be that some non-specialized HCPs and allied caretakers not fully understand and recognize the massive caregiving provided by parents, and how this affects the whole family. Due to this, the parents could lack the support and help they need. HCPs should have a holistic approach in caring for individuals with EB, and recognize the practical and emotional burdens these parents meet.

## Electronic supplementary material

Below is the link to the electronic supplementary material.

Supplementary Material 1

## Data Availability

The datasets generated and/or analysed during this study are not publicly available due its sensitive nature. Data supporting the findings of the current study are not publicly available but only stored in Centre for rare Disorders, in an approved research information system, maintaining adherence with OUS policy, and local ethics requirements.
