# Supplementary material for: When care hurts: parents’ experiences of caring for a child with epidermolysis bullosa
Source: Orphanet J Rare Dis. 2024 Dec 27;19:492. doi: 10.1186/s13023-024-03502-5 (PMC11681652; doi:10.1186/s13023-024-03502-5)
Supplement: Supplementary file 1 — Supplementary Material 1 [file 13023_2024_3502_MOESM1_ESM.docx]

**Interview guide**

Could you please tell me a little bit about your motivation to participate in this study?

How old are you?

What kind of work do you do?

Who do your closest family members consist of?

Is there more than one affected by EB in the family? Which subgroup is your child diagnosed with?

**Pregnancy/birth**

Could you please tell me about your (your wife’s) pregnancy and birth?

**Diagnostic process**

Could you please tell me about the diagnostic process and how you experienced it?

**Coming home from hospital after birth**

Could you please tell me how you experienced coming home from hospital after birth?

**Feelings toward the child**

Could you please tell me about your feelings toward the child?

Could you please tell me how the child signaled pain/discomfort and how easy it was for you to understand the child’s signals?

**Skin care**

Do you remember the first skin care? Could you please tell me about it?

Could you please tell me about how you experienced touching the child’s skin?

Tell me about how you prepared the child and yourself for skin care at different ages, please.

**Everyday life**

Could you please tell me what a normal day looked like when you came home from the hospital?

Could you please tell me how you cuddled the child?

How easy was it for you to understand the child’s communication?

Could you please tell me how you experienced being both a parent and responsible for skin care?

Did you experience that the parental role was affected by the fact that the child was born with EB?

Was there anything you would like to do together with your child that was difficult because of EB?

What worried you the most?

How did you feel about carrying out skin care when the child grew and became more mobile?

**Social experiences**

Did you get any questions from other people about what was wrong with your child? If yes, could you please tell me how you answered those questions?

Do you experience that the child has met any negative social reactions due to EB?

Do you think EB affects your child’s relationships with other people?

**Attachment**

Do you think EB has affected your attachment to your child?

**Closing comment**

Is there anything I haven’t asked but that is important to you?
